# Effect of temperature on the CO_2_ splitting rate in a DBD microreactor

**DOI:** 10.1039/d3re00113j

**Published:** 2023-05-09

**Authors:** Deema Khunda, Sirui Li, Nikolay Cherkasov, Mohamed Z. M. Rishard, Alan L. Chaffee, Evgeny V. Rebrov

**Affiliations:** a School of Engineering, University of Warwick Coventry CV4 7AL UK E.Rebrov@warwick.ac.uk; b Department of Chemical Engineering and Chemistry, Eindhoven University of Technology Eindhoven The Netherlands E.Rebrov@tue.nl; c The University of Auckland Auckland New Zealand; d School of Chemistry, Monash University Melbourne Victoria Australia

## Abstract

A novel plate-to-plate dielectric barrier discharge microreactor (micro DBD) has been demonstrated in CO_2_ splitting. In this design, the ground electrode has a cooling microchannel to maintain the electrode temperature in the 263–298 K range during plasma operation. A small gap size between the electrodes of 0.50 mm allowed efficient heat transfer from the surrounding plasma to the ground electrode surface to compensate for heat released in the reaction zone and maintain a constant temperature. The effect of temperature on CO_2_ conversion and energy efficiency was studied at a voltage of 6–9 kV, a frequency of 60 kHz and a constant CO_2_ flow rate of 20 ml min^−1^. The CO_2_ decomposition rate first increased and then decreased as the electrode temperature decreased from 298 to 263 K with a maximum rate observed at 273 K. Operation at lower temperatures enhanced the vibrational dissociation of the CO_2_ molecule as opposed to electronic excitation which is the main mechanism at room temperature in conventional DBD reactors, however it also reduced the rate of elementary reaction steps. The counterplay between these two effects leads to a maximum in the reaction rate. The power consumption monotonously increased as the temperature decreased. The effective capacitance of the reactor increased by 1.5 times at 263 K as compared to that at 298 K changing the electric field distribution inside the plasma zone.

## Abstract 2

Ground electrode cooling improved CO_2_ conversion in a DBD microreactor. A maximum in CO_2_ conversion of 10.6% was observed at a gas temperature of 363 K.

## Graphical abstract

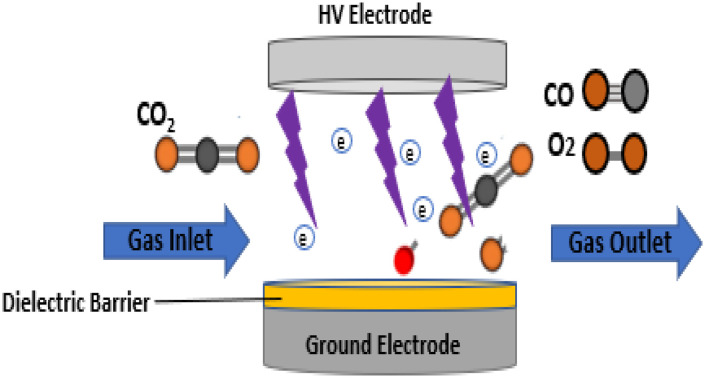

## 1. Introduction

Carbon capture and utilization (CCU) has emerged as an effective method for reducing global greenhouse gas emissions and has been established as a central research topic for the past decade. CCU is recognized as an optimal route to process CO_2_ gas compared to carbon capture and storage (CCS). It allows effluent CO_2_ gas from chemical processes to dissociate in a renewable route fully independent from fossil fuels.^1,2^ Utilization of CO_2_ gas can be achieved by several approaches, such as thermal decomposition, electrocatalysis, solar-to-chemical methods, and plasma. However, each approach presents particular challenges; for example, thermal decomposition requires high temperatures (>2000 K) drastically decreasing energy efficiency while electrocatalysis and photocatalysis typically require expensive noble metal catalysts.^3^ Of all these approaches, non-thermal plasma (NTP) showed promising potential for utilizing CO_2_ gas at high efficiency.^4^ NTP provides a cost-effective way for thermodynamically unfavourable reactions to occur at low temperatures and pressures, offering advantages over other CO_2_ processing routes.^5,6^ Despite these advantages, the technology faces challenges in improving efficiency, CO_2_ conversion, and reducing energy costs.^4^

Recent research in CO_2_ splitting under NTP plasma conditions has explored a wide range of parameters to enhance conversion and efficiency, such as gas flow rate, input power, operational frequency, voltage, and the size of discharge gap. Various discharge types can be used for CO_2_ conversion in plasma, including dielectric barrier discharges (DBD), gliding arc, microwave, and nanosecond pulses. DBD is a typical example of cold plasma where the CO_2_ gas remains at near room temperature,^7^ while gliding arc and microwave plasma are classified as warm plasmas where the gas temperature can reach up to 1000 K.^8^ Bogaerts *et al.*^9^ found that lower flow rates increase conversion but result in lower energy efficiency, achieving a maximum efficiency of 15%, while narrow discharge gaps increase efficiency achieving 10% at a CO_2_ conversion of 30%.^10^ Prior studies utilized supported transient (*e.g.* Ni/Al_2_O_3_)^11^ and noble metal (*e.g.* Rh/Al_2_O_3_)^12^ catalysts to target production of specific products such as acetic acid and hydrocarbons. It was reported that adjusting plasma parameters enhances the synergistic interaction between the plasma and the catalyst to improve the reaction rate. Despite significant research aimed at improving DBD performance, energy efficiency remains below the threshold value to compete with other CO_2_ processing technologies.^13^ In fact, in plasma-assisted CO_2_ conversion there is an interplay of many effects: reactive species transport, complex gas phase chemistry, and electromagnetic field, which are not well understood, but they are important for optimization of the reactor performance.^8^ Electrochemical conversion has already achieved a solar-to-fuel efficiency of 19%, defined as the energy stored in the fuel to the energy input.^14,15^ Therefore at least a similar level of overall energy efficiency is needed for NTP technology to be competitive. This means an energy efficiency of at least 60% is needed for the plasma step in syngas production from CO_2_ to reach this overall target.

Precise temperature control is a possible way to enhance the energy efficiency and CO_2_ conversion in DBD reactors. Investigations into the effect of gas temperature are still scarce, with only a few studies employing forced gas cooling to enhance the performance of DBD reactors.^16,17^ When active cooling was employed, a burst mode, a rapidly pulsed power regime for DBDs, was found to provide only minor benefits or no benefit, compared to a continuous mode of operation.^16^ Zhu *et al.*^17^ reported that CO_2_ conversion reached 49% at an energy efficiency of 10% over foamed Ni and Cu meshes when the gas was cooled. In atmospheric pressure DBD plasma, Xi *et al.*^18^ found that the gas temperature affects the O_3_ concentration. They compared the results for the gas temperatures of 5 and 50 °C, and noted that the concentration of O_3_ decreases at the higher temperature at a constant discharge power. Several cooled DBD reactors were investigated under a periodic operation rather than a continuous mode of operation.^19,20^ It is evident from the above that the gas temperature can indeed affect the changes in chemical product distribution. However, the change in power influences the gas temperature, and therefore affects the species density, thereby leading to variation in the reduced electric field (the electric field divided by the background gas number density).

Modelling insights revealed that higher energy efficiency values at lower gas temperatures were due to the promotion of vibrational excitation (VE) of CO_2_ in DBD reactors.^21^ The CO_2_ molecule dissociates in a ladder-climbing process in which electrons gradually populate the higher vibrational levels to end in CO_2_ dissociation.^4,22^ VE is reported to decrease the activation energy of the dissociation reaction.^23^ A lower gas temperature allows a larger number of molecules to dissociate by VE than by other mechanisms. In fact, VE assisted dissociation is the main mechanism in microwave and gliding arc plasmas achieving higher energy efficiency as compared to DBD. In microwave plasma, 90% of electron energy is consumed by VE as opposed to 10% in DBD.^4^ Cooling the gas reduces the reduced electric field^24^ and this, in turn, results in more efficient CO_2_ dissociation. This effect can be achieved by increasing the heat transfer rate at higher flow rates,^25^ reducing the gap size between the electrodes, increasing the electrode surface area or increasing the temperature gradient between the electrode temperature and the surrounding gas temperature.^26^

In this paper, we investigate the effect of gas temperature on CO_2_ conversion and energy efficiency in a DBD microreactor. The application of plasma in a microreactor is an attractive tool for studying plasma flow chemistry and process intensification, allowing precise control of reaction conditions.^27,28^ Plasma was generated in a 0.50 mm gap providing a higher surface area-to-volume ratio to enhance heat transfer. The ground electrode temperature was varied in the range below room temperature, while the temperature of the second (high voltage) electrode remained close to the gas temperature. Forced cooling in the microreactor is studied to enhance energy efficiency and conversion, paving the route for DBD reactors to be used for processing CO_2_ gas.

## 2. Experimental & methodology

### 2.1 Microplasma reactor

**Fig. 1:**
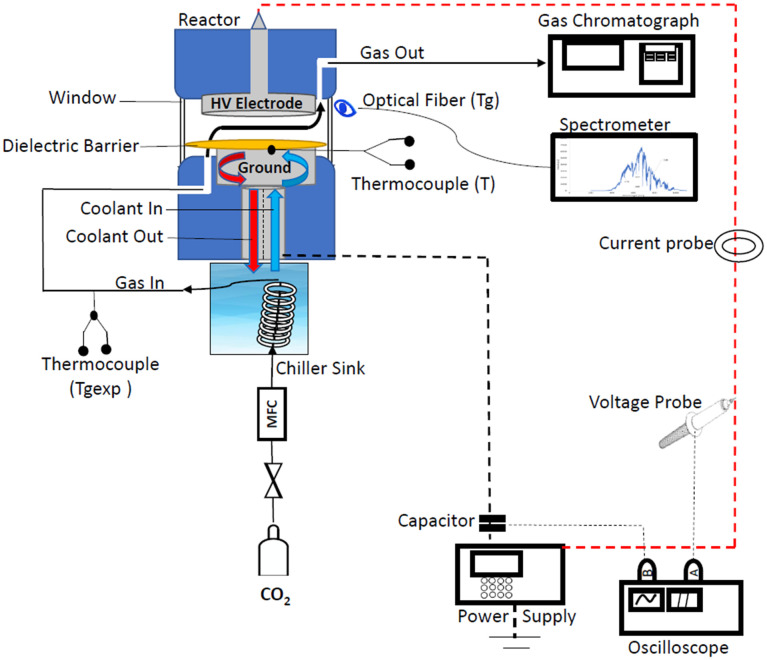
Schematic diagram of the experimental set-up.

The experiments were conducted in a plate-to-plate DBD microreactor as shown in Fig. 1. A titanium disc with a diameter of 24.4 mm was used as the high voltage (HV) electrode. The ground electrode was made of a brass disk with a diameter of 19.5 mm. The ground electrode has a cooling channel with a diameter of 0.20 mm. A dielectric Mica film layer (dielectric constant of 7) with a thickness of 0.20 mm was deposited on the ground electrode. The electrodes were positioned in a PEEK (polyetheretherketone) housing at a distance of 0.5 mm from each other.

A mixture of ethylene glycol and water was pumped *via* the ground electrode during operation at a desired temperature. The CO_2_ gas (99.99 vol%) was cooled in a thermostat (Lauda) and then fed to the reactor at a flow rate of 20 ml min^−1^ (STP) with a mass flow controller (Bronkhorst). The power input to the DBD reactor was controlled by changing the peak-to-peak voltage of the signal generated by a power generator (G2000 Redline Technologies). A high voltage probe (P6015A ground-referenced 100 MΩ 3.0 pF) was used to measure the voltage across the reactor, and it was connected to an oscilloscope (PicoScope) to record voltage waveforms. The current waveforms were measured by a current probe (Pearson Electronics). An external capacitor (400 pF) was connected between the ground electrode and the oscilloscope. Nitrogen was added to the gas mixture after the plasma reactor and it was used as an external standard. The gas outlet flow was fed to a gas chromatograph (Shimadzu 2010 GC) equipped with a FID and TCD. The optical emission spectra were recorded with a spectrometer with an integrated deep controlled back-illuminated CCD detector (slit width 25 μm, FERGIE, Princeton Instruments). The OES spectrometer was connected to the reactor *via* an optical fiber.

Experiments were conducted by varying the applied voltage at several ground electrode temperatures. All the experiments were performed at least twice (reproducibility was above 95%). The temperatures of the plasma region and the gas outlet were monitored with thermocouples. The electrode temperature (*T*_c_) was measured by a thermocouple attached to the electrode surface as a function of coolant temperature and the flow rate in the absence of plasma. At the same time, the gas temperature was measured by a thermocouple positioned at the reactor outlet (*T*_gexp_). Several electrode temperatures in the 263–293 K range were studied at applied voltages in the range between 6.0 and 9.3 kV. The electrode temperature remained stable around the setpoint value within a ±0.5 K range during experiments. The experiments were only started after the electrode temperature measured by the thermocouple has reached the setpoint value and remained stable for a period of 10 min. The gas, coolant tubes and ground electrode were thermally insulated to prevent heat flux from the environment while the electrode temperature decreased from 293 to 263 K.

The AC frequency and the gas flow rate were maintained at 60 kHz and 20 ml min^−1^ respectively in all experiments. Table 1 shows the experimental conditions leading to stable discharges. Some conditions produced either no stable discharge or no discharge at all. In general, a lower electrode temperature requires a higher voltage to achieve gas breakdown in agreement with ref. 29.

**Table 1:** Experimental conditions leading to stable discharges

*T* (K)	Voltage (kV)					
	6.0	6.5	7.0	7.6	8.7	9.3
263	—	—	—	✗	✗	✗
268	—	—	—	✗	✗	✗
273	—	—	✗	✗	✗	*
278	—	—	✗	✗	✗	*
293	—	✗	✗	✗	✗	*

### 2.2 Gas conversion and power consumption

The CO_2_ conversion was calculated by eqn (1). Molar flow rates were used instead of volumetric flow due to the expansion of the number of moles in the course of reaction.^29^1
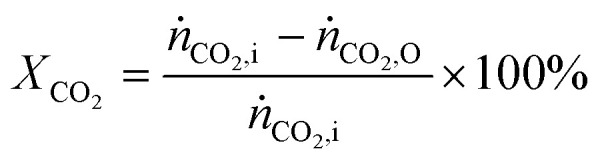
where *ṅ*_CO_2_,i_ and *ṅ*_CO_2_,O_ are the CO_2_ inlet and outlet molar flow rates, respectively. The energy efficiency was calculated based on theoretical energy requirement *vs.* actual energy consumed in the reactor:2
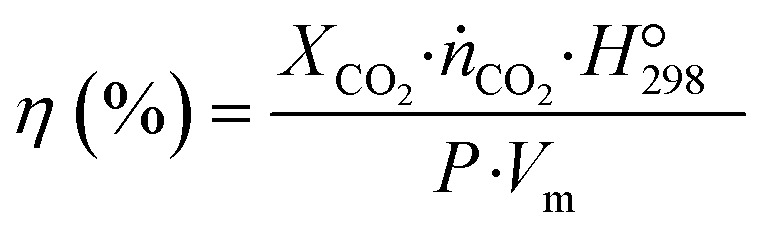
where 
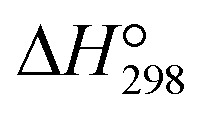
 is the standard reaction enthalpy of CO_2_ splitting (283 kJ mol^−1^), and *V*_m_ is the molar volume under standard conditions, 22.4 L mol^−1^. The total power (*P*) was calculated *via* the Lissajous method:3
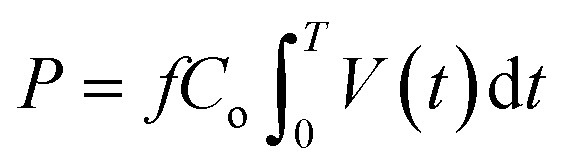
where *f* is the frequency of the AC electric field, *C*_o_ is the capacitance of the capacitor (400 pF), *V*(*t*) is the voltage applied to the reactor, and *T* is the time corresponding to one period of voltage change. In all experiments, CO and O_2_ were the only products observed.

### 2.3 Gas temperature measurements with OES

The gas temperature was estimated during plasma operation by the Boltzmann plot method and compared with the electrode temperature. The method is widely used for temperature measurements of plasma gas discharges.^30–32^ The Boltzmann distribution gives a relationship between the emission intensities (*I*_*J*_) of the rotational lines and the rotational temperature (*T*_r_):^31^4
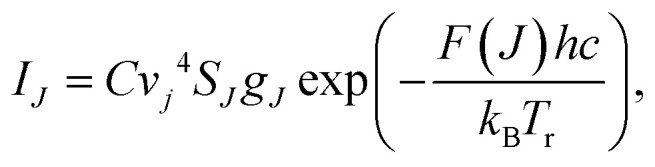
where *J* is the rotational quantum number, *C* is a constant independent of *J*, *g*_*J*_ is the degeneracy of the rotational level *J* which is given by (2*J* + 1), *v*′ is the vibrational frequency of the transition, *c* is the speed of the light, and *h* and *k*_B_ are the Planck and Boltzmann constants, respectively.

Optical emission spectra (OES) of CO_2_ plasma were recorded at a voltage of 8.3 kV at two electrode temperatures of 273 and 293 K. The lines of the Q branch of the 481 nm peak (B^1^Σ^+^(*v*′ = 0) → A^1^Π, *v*′′ = 1) were taken at *J* = 9–22 for calculation of rotational temperature (*T*_r_). Then eqn (5) was used to calculate the gas temperature (*T*_g_):^33^5
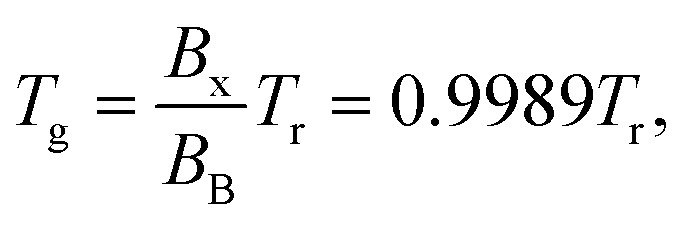
where *B*_x_ and *B*_B_ are the rotational constants of the ground (X^1^S) and the excited (B^1^Σ^+^) states of the CO molecule. The variation of the vibrational frequency *v* was quite small across the Q-branch and it was assumed to be constant. Under such assumption, the rearrangement of eqn (4) gives:6
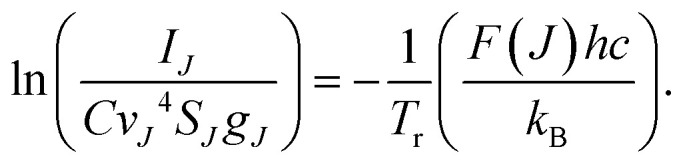
Therefore, the left hand side of eqn (6) is directly proportional to *F*(*J*), and the slope is proportional to the inverse of rotational temperature (*T*_r_). The term *F*(*J*) represents the energy of the rotational levels of the upper electronic state:^32^

7 *F*(*J*) = *B*_v_*J*(*J* + 1) − *D*_v_*J*^2^(*J* + 1)^2^,

where *B*_v_ is the rotational constant for the B^1^Σ^+^ (*v*′ = 0) state (*B*_v_ = 1.94808 cm^−1^) and *D*_v_ is the centrifugal distortion (*D*_v_ = 6.33 × 10^−6^ cm^−1^).^34^ The Honl–London factor (*S*_*J*_) for the B^1^Σ^+^ (*v*′ = 0) → A^1^Π (*v*′′ = 1) transition is given by eqn (8):8
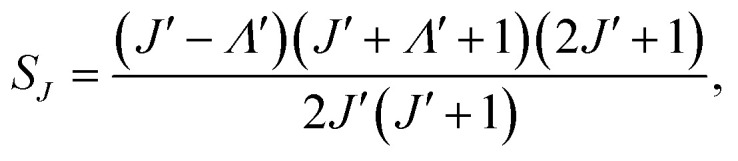
where *Λ*′ = 0 for the B^1^Σ^+^ state.

## 3. Results and discussion

### 3.1 Effect of temperature on plasma

**Fig. 2:**
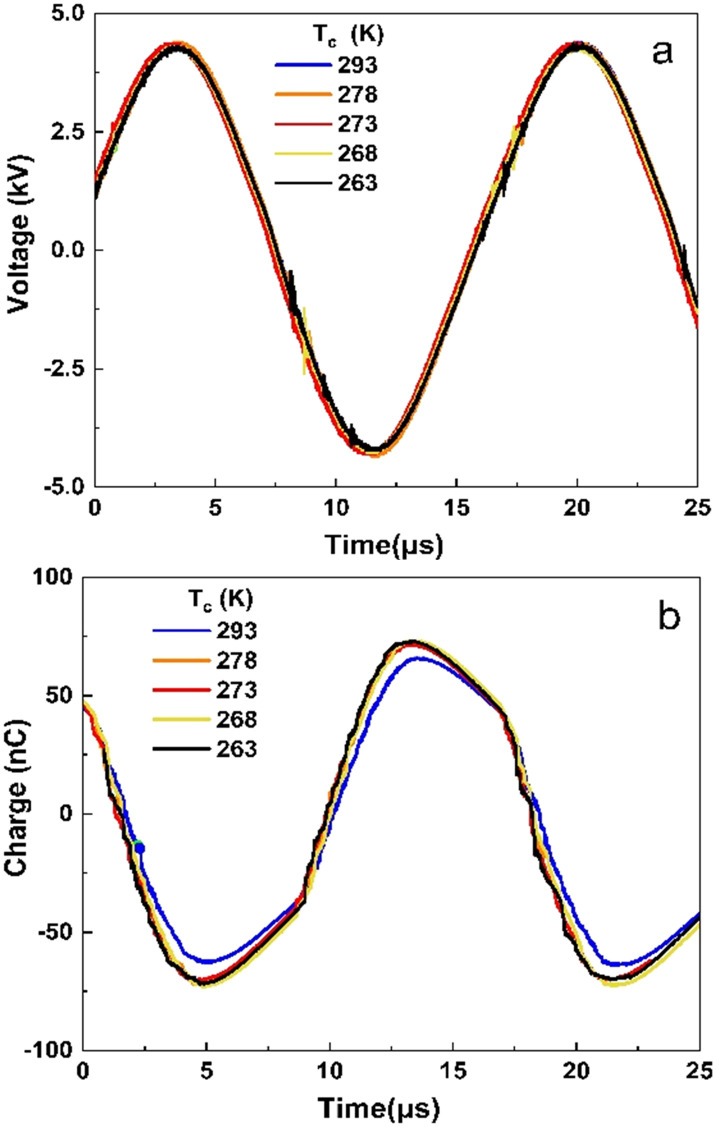
(a) Voltage in a DBD reactor and (b) charge as a function of time at five temperatures. Discharge gap: 0.50 mm, CO_2_ flow rate: 20 ml min^−1^, voltage: 8.7 kV, frequency: 60 kHz.

**Fig. 3:**
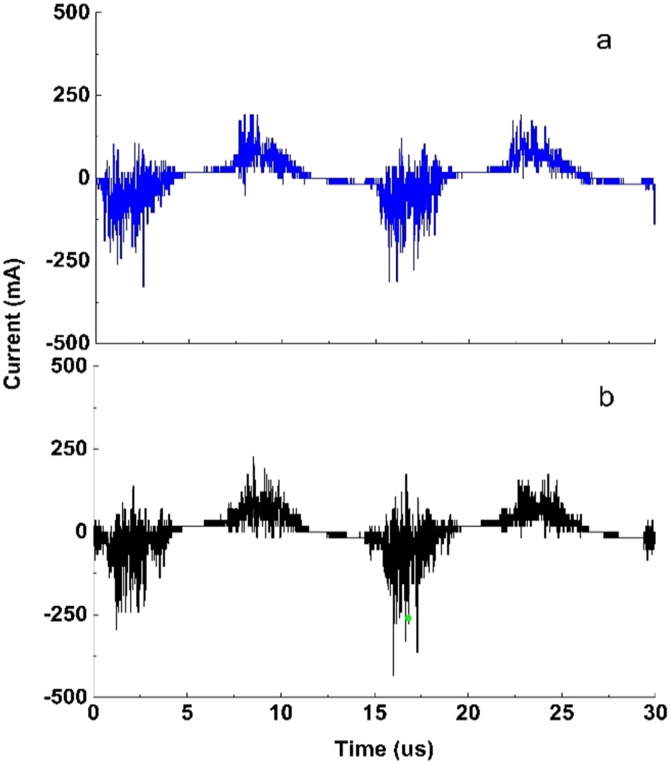
Current waveforms at a temperature of (a) 263 K and (b) 293 K. Other input parameters are the same as those in Fig. 2.

Fig. 2a and b show the voltage and charge waveforms at different electrode temperatures and an applied voltage of 8.7 kV. Fig. 2(a) shows less than 0.1% variability in applied voltage between the different temperatures. However, the charge transferred was affected by the temperature, with the lower temperature resulting in higher charge dissipation (Fig. 2b). The maxima in the charge waveform at 263 K are higher than those at 293 K. This indicates that power dissipation increases as the temperature decreases. The current waveforms are shown in Fig. 3a and b for 263 and 293 K, respectively. It can be concluded that the reactor is in the filamentary discharge mode characterized by transient micro discharges between the two electrodes.

**Fig. 4:**
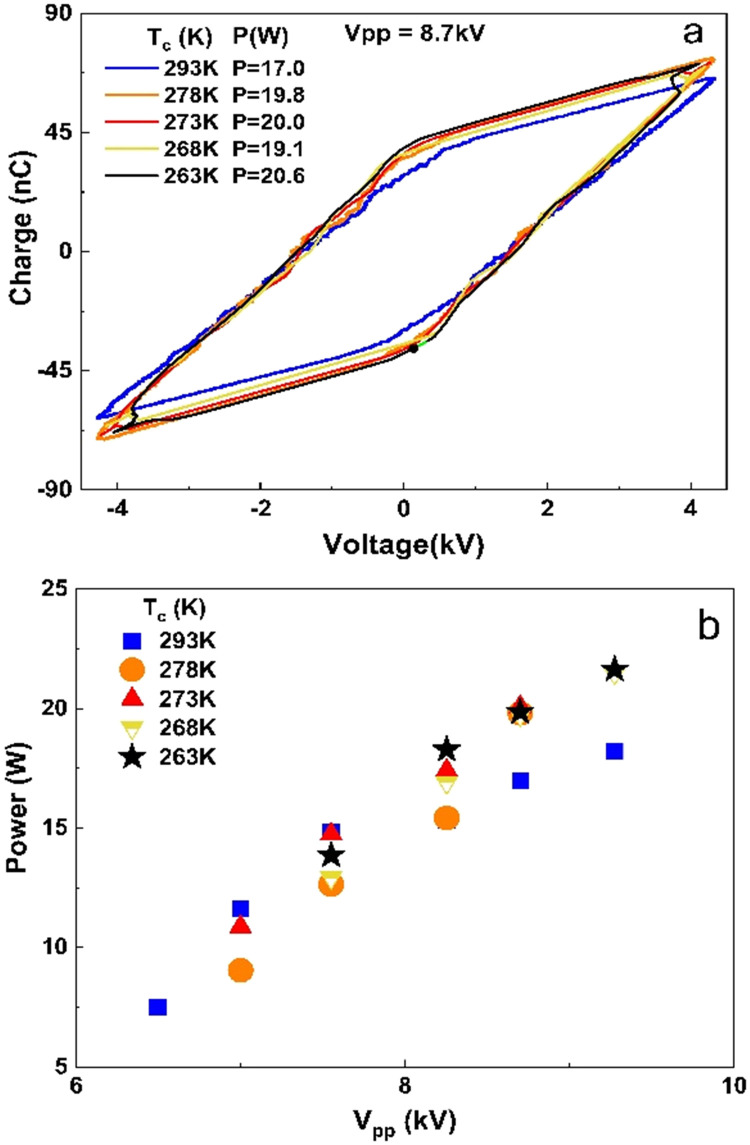
(a) Lissajous figure at 8.7 kV at several electrode temperatures. (b) Power dissipated at several electrode temperatures. Experimental conditions are the same as those in Fig. 2.

The Lissajous figure is presented in Fig. 4(a) for each temperature at a voltage of 8.7 kV. The area between the curves is larger at a temperature of 263 K as compared to 293 K. A larger area of the Lissajous figure indicates that more micro-discharges are taking place, leading to a greater chance of reaction with plasma species and more energy being used for CO_2_ decomposition.^35^ The power consumption increases from 17.0 to 20.6 W as the temperature decreases. Fig. 4b demonstrates power consumption against applied voltage. It can be seen that higher power consumption was observed at lower temperatures in the whole range of voltages studied.

**Fig. 5:**
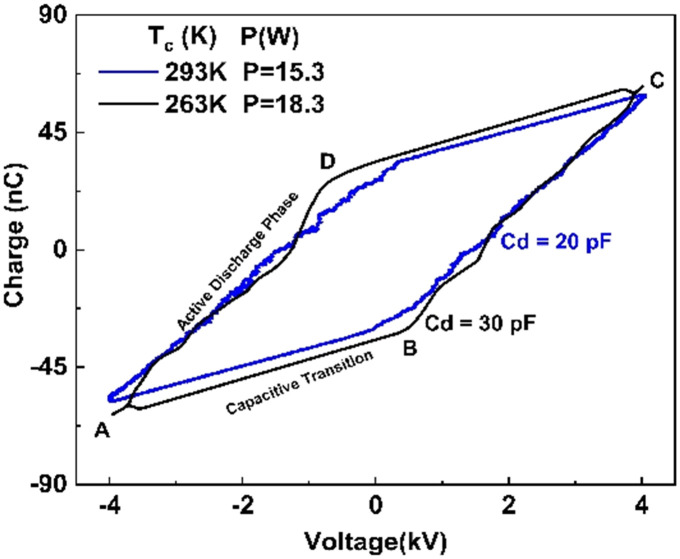
Lissajous figure at a voltage of 8.3 kV (peak to peak) at 263 and 293 K. Lines AB and CD represent the discharge-off phase when there is only a displacement current and their slopes correspond to the cell (*C*_cell_) in the plasma-off period. Lines BC and DA represent the discharge-on phase when the gas breakdown occurs in the gap and the plasma is ignited. The slope of these lines is effective capacitance (*C*_d_). Experimental conditions are the same as those in Fig. 2.

To closely examine plasma behaviour, the Lissajous plots at an applied voltage of 8.3 kV are shown in Fig. 5 at two temperatures of 263 and 293 K. As the temperature decreases from 293 to 263 K, the area slightly increases, while the reactor effective capacitance (*C*_d_) during discharge increases 1.5-fold, from 20 to 30 pF. As the effective capacitance increases more breakdown of gas is achieved. This is confirmed by the increase in slope at the lower temperature during the active discharge phase suggesting that the reactor approaches uniform discharge behaviour and exhibits less filamentary discharge behaviour which is associated with higher transferred charges during breakdown.^35^

The dielectric constant of the barrier layer measures the electric polarizability of the reactor. With a higher dielectric constant, *i.e.* higher relative permittivity, the material polarizes more to the applied electric field. This is an important parameter in DBD reactors as it may influence both CO_2_ conversion^35^ and energy efficiency.^36^ The breakdown strength of dielectrics generally increases at lower temperatures due to reduced dipole mobility.^37^ However the dielectric mica film was shown to have little variation with temperature. A breakdown strength of mica of 1.4 × 10^7^ V cm^−1^ was virtually independent of temperature below 100 K (ref. 38) and varies only within 1.5% in the 300–500 K range.^39^ Therefore, the difference in breakdown voltage between 278 and 293 K can be explained by the effect of gas temperature rather than the change in the dielectric properties of the mica layer.

### 3.2 Effect of temperature on breakdown voltage

**Fig. 6:**
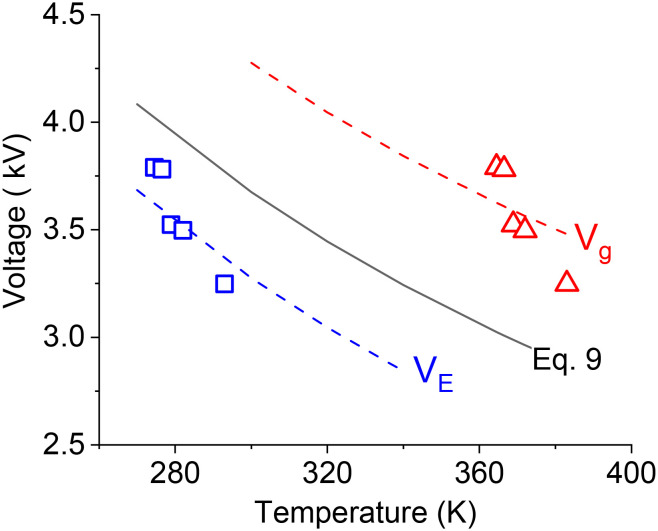
Theoretical CO_2_ breakdown voltage calculated by eqn (9) and experimental breakdown voltage based on the electrode surface temperature (*V*_E_) and gas temperature (*V*_g_)_._

The breakdown voltage (*V*_b_) is higher at lower temperature which is consistent with a previous study^40^ where breakdown voltage was found to be inversely proportional to gas temperature:9
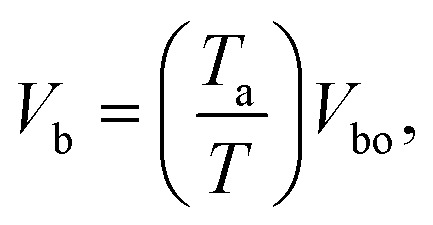
where *T*_a_ is the ambient temperature and *V*_bo_ is the breakdown voltage at ambient temperature, which is estimated by empirical eqn (10):^41^10
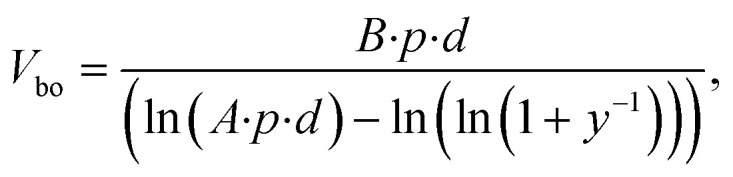
where, *p* is the pressure, *d* is the gap distance between the electrodes, *γ* is the secondary electron emission coefficient of the electrode material, and *A* and *B* are the empirical constants for each gas.^42^ Although eqn (10) mainly applies for the case of two metal electrodes, it can still be used as a qualitative tool for obtaining breakdown voltages in DBD reactors. Eqn (10) suggests that the breakdown voltage for CO_2_ at a temperature of 293 K is equal to 3.8 kV, while the experimentally measured breakdown voltage is 3.25 kV (*V*_pp_ = 6.5 kV). Fig. 6 compares the experimental (*V*_E_) breakdown voltages and those calculated by eqn (9) at different electrode temperatures. It can be seen that the experimental values are below the theoretical predictions which is an indication that the actual gas temperature is considerably higher than the electrode temperature.

Therefore, in the next step, the gas temperature was determined by optical emission spectroscopy following the method described in section 2.3.

### 3.3 Gas temperature measurement by optical emission spectroscopy

**Fig. 7:**
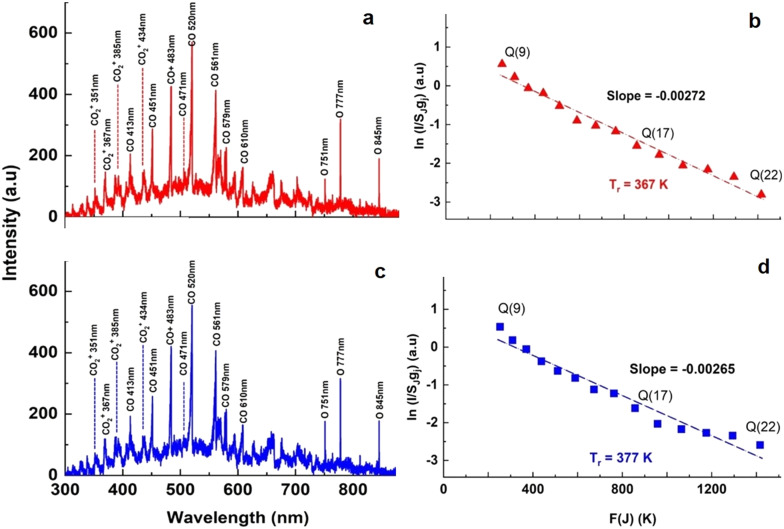
OES spectra of CO_2_ plasma at a power of 15 W and an electrode temperature of (a) 273 and (c) 293 K. The corresponding Boltzmann plots of the selected lines of the Q branch of the Angstrom band of CO (0–1) at (b) 273 K (d) 293 K.

The optical emission spectra of CO_2_ plasma are shown in Fig. 7a and c at the electrode temperatures of 273 and 293 K, respectively. Several CO bands were identified and their positions are listed in Table 2. The CO Ångström system (B^1^Σ^+^ → A^1^Π) between 450 and 650 nm (ref. 30) was observed at both temperatures. The bands of excited CO_2_^2+^ (Fox–Duffendack–Barker system) were also observed. Fig. 7b and d show the corresponding Boltzmann plots. The rotational gas temperatures, obtained by eqn (6), were 367 and 377 K, respectively. Eqn (5) suggests that the bulk gas temperature is essentially the same as the rotational temperature. While the absolute gas temperature is considerably higher than the electrode temperature, the temperature difference between the gas temperature and electrode temperature remains relatively constant and is equal to 94 and 84 K for these two cases. Such a difference can be explained by a relative error of about 5 K in estimation of rotational temperature (*R*^2^ = 0.97). This allows estimation of the heat loss *via* the electrode surface using Newton's law of cooling for laminar flow between two parallel plates. While the second electrode was not cooled during plasma operation, it can be assumed that all heat is removed *via* the surface of the ground electrode. This disturbs the ideal case and therefore the actual heat transfer coefficient may be slightly lower as compared to the case of symmetrical cooling from both sides. Anyway, it provides a conservative estimation for the heat transfer rate from the bulk plasma zone towards the coolant. It needs to be mentioned that the conduction thermal resistance in the Cu electrode and convective thermal resistance in the cooling microchannel are much less than the convective resistance in the gas boundary layer around the electrode surface. Therefore the overall heat transfer coefficient is equal to the convective heat transfer coefficient in the gas boundary layer. It is known that at a constant wall temperature, the heat transfer coefficient depends only on the geometry of the channel cross section and the corresponding Nu number is equal to 7.54 for parallel plate geometry. Then the heat removal rate can be calculated by eqn (11).

11 *Q* = *hA*(*T*_g_ − *T*_S_),

where *h* is the heat transfer coefficient, calculated from the corresponding Nu number, *A* is the ground electrode surface area (*A* = π*d*^2^/4 = 2.99 × 10^−4^ m^2^), *T*_g_ is the bulk gas temperature measured by OES, and *T*_S_ is the electrode temperature measured by the thermocouple.

**Table 2:** Species identified in the OES and their respective electronic transitions and intensities^31^

Species	Wavelength (nm)	Transition	Peak intensity	
			293 K	273 K
CO_2_^+^	351	FDB, A^2^Π → X^2^ ((*υ*′, 0, 0) → (*υ*′′, 0, 0))	74	95
			94	78
	367		77	81
	385			
CO_2_^+^	434.2	FDB, A^2^Π → X^2^ ((*υ*′, 0, 0) → (*υ*′′, 0, 2))	88	96
CO	471.7	Triplet system, *d*^3^Δ → *a*^3^Π	66	76
CO	451	Ångström system, B^1^Σ^+^–A^1^Π (0–0)^a^	258	288
	483	B^1^Σ^+^–A^1^Π (0–1)	421	426
	520	B^1^Σ^+^–A^1^Π (0–2)	555	549
	561	B^1^Σ^+^–A^1^Π (0–3)	407	408
	579	B^1^Σ^+^–A^1^Π (0–4)	216	227
	610	Ångström system	164	162
O	751	3p^5^P → 3s^5^S^0^	177	124
	777		316	305
			178	190
	845			

a The Angstrom system of CO from the electronic transition of B^1^Σ^+^ → A^1^Π of CO is attributed to the *v*′ = 0 vibrational level of the upper electronic state to vibrational levels (*v*′′ = 0, 1, 2, 3, and 4) of the lower electronic state.

The analysis performed using eqn (11) gives a heat transfer rates of 15.0 and 15.1 W for electrode surface temperatures of 273 and 293 K, respectively. There is one more term which needs to be considered in the overall heat balance in the reactor, which is related to the heating of the gas stream from the inlet temperature (*T*_in_) to the mean bulk temperature (eqn (12)):

12 *Q* = *ṁC*_p_(*T*_g_ − *T*_in_),

where *C*_p_ is the mean gas heat capacity and *ṁ* is the mass flow rate. However due to a very low CO_2_ mass flow rate in the reactor, its contribution remains less than 1% of the total heat losses. Thus the heat generation rate by plasma is in a good agreement with the heat removal rate *via* forced convection. Based on these data, it can be concluded that the rather accurate prediction of mean gas temperature in the micro DBD reactor was obtained by OES. The cooling of the electrode surface together with its high surface to gas volume ratio is able to maintain the gas temperature just 90 K above the electrode temperature.

### 3.4 Effect of temperature on CO_2_ conversion and energy efficiency

**Fig. 8:**
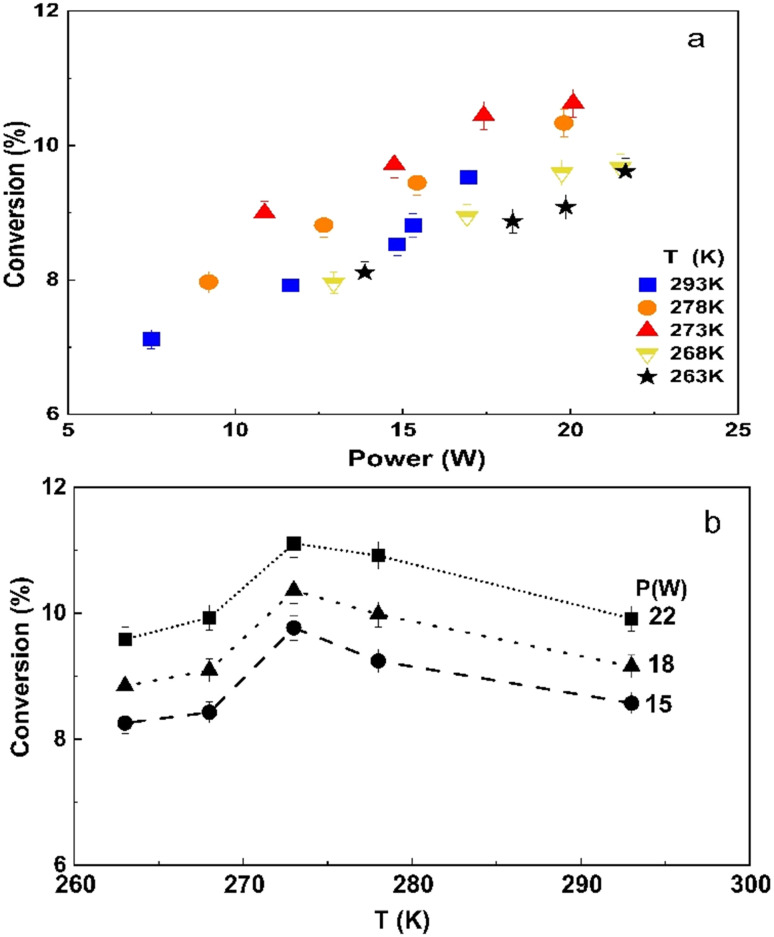
CO_2_ conversion as a function of (a) power at different temperatures and (b) temperature at different input powers.

The CO_2_ conversion gradually increases with discharge power in the entire temperature range Fig. 8a, as higher power increases the electron density and the rate of electron collision with CO_2_ molecules.^43^ The conversion increases as the temperature decreases from 293 to 273 K. However, a 2-fold increase in input power results in just a moderate (20 to 30%) improvement in the reaction rate in this temperature range. The CO_2_ conversion at 273 K demonstrates a maximum value in the entire range of input powers studied as it can also be seen in Fig. 8b when replotting the conversion data as a function of temperature. The conversion generally increases inversely proportionally to the temperature in the 273–293 K range. For example, at a power of 15 W, the CO_2_ conversion starts at 8.6% at room temperature and increases to 9.7%, when the temperature decreased to 273 K. Multiple factors can explain this trend. Firstly, the rate of recombination reactions decreases, “freezing” the products.^44^ Secondly, more energy transfers to the vibrational levels of CO_2_, initiating the vibrational ladder climbing process rather than electronic excitation, while the latter is a less efficient mechanism for CO_2_ dissociation.^45^ The lifetime of vibrationally excited species also increases at lower temperatures.^44,46,47^ Lu *et al.*^48^ demonstrated that the DBD operation at 20 W and at a constant temperature of 313 K enhanced CO_2_ conversion and resulted in a 6-fold improvement of energy efficiency compared to the operation without external cooling.

However, it is important to note that CO_2_ decomposition is an endothermic reaction, and therefore the equilibrium is shifted towards the products as the temperature increases. Therefore, the positive effect of the vibrational ladder climbing process is counteracted by the reduced rate of elementary reaction steps in the temperature range below the electrode temperature of 273 K (or gas temperature below 363 K). As a result, the CO_2_ conversion decreases, showing an optimum electrode temperature of around 273 K in the DBD microreactor.

The main reaction pathways for CO_2_ dissociation in plasma is represented by two routes: (1) electron impact dissociation to CO and O [reaction (1)] and (2) electron impact ionization to CO_2_^+^ [reaction (2)]:^49^

R1 e^−^ + CO_2_ → CO + O + e^−^

R2 e^−^ + CO_2_ → CO_2_^+^ + e^−^ + e^−^

Once the ionization process begins in reaction (R2), the CO_2_ molecule's interaction with ionized CO_2_^+^ to produce C_2_O_4_^+^ ions reaction (R3) becomes the main reaction pathway. The C_2_O_4_^+^ ions interact with CO to produce C_2_O_3_^+^reaction (R4), and C_2_O_3_^+^ interacts further with CO to produce C_2_O_2_^+^reaction (R5). C_2_O_2_^+^ can either react with free electrons to result in two CO molecules reaction (R6) or interact, releasing energy and resulting in CO and CO^+^reaction (R7). These reactions are listed below:^50^

R3 2 × (CO_2_^+^ + CO_2_ + M → C_2_O_4_^+^ + M)

R4 2 × (C_2_O_4_^+^ + CO + M → C_2_O_3_^+^ + CO_2_ + M)

R5 2 × (C_2_O_3_^+^ + CO + M → C_2_O_2_^+^ + CO_2_ + M)

R6 e^−^ + C_2_O_2_^+^ → CO + CO

R7 C_2_O_2_^+^ + M → CO + CO^+^ + M

R8 CO^+^ + CO_2_ → CO + CO_2_^+^

All these ionization reactions consume CO and produce CO_2_ in the process; once the full circular pathway interaction is complete, the net conversion of CO_2_ into CO is mainly achieved by the electron impact dissociation reaction (R9) represented below:

R9 3e^−^ + CO_2_ → CO + O + 3e^−^

Alliati *et al.*^51^ conducted a chemical kinetic model study and found that CO_2_ dissociation by electron impact reactions was responsible for 95% of conversion, while electron attachment contributes to the remaining 5%. This result is in qualitative agreement with Ponduri *et al.*^52^ where 80% of conversion was due to electron impact reactions.

**Fig. 9:**
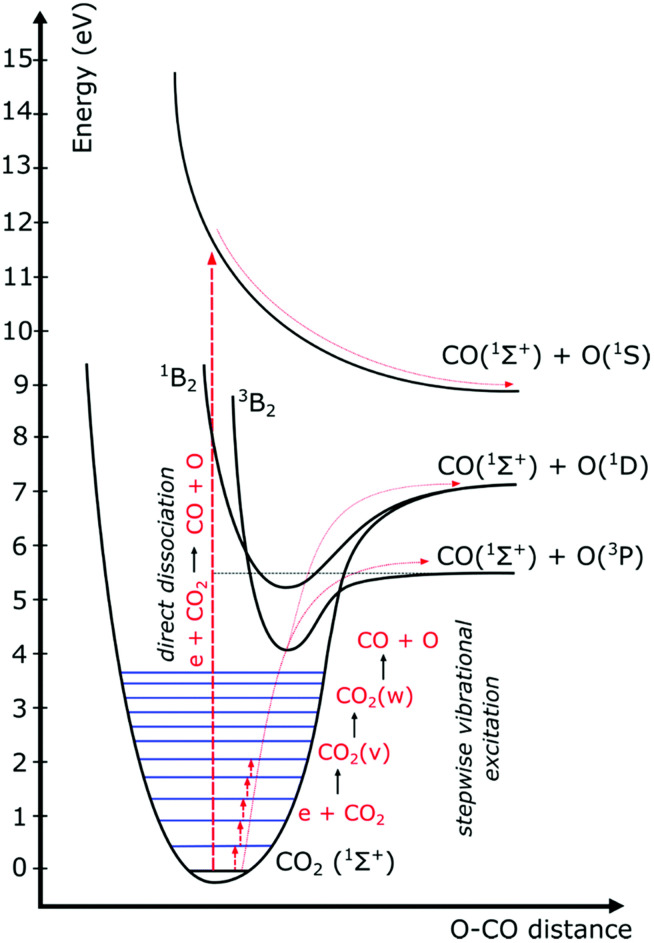
Schematic diagram of vibrational and electronic excitation levels of the CO_2_ molecule (reproduced from ref. 53 with permission from the Royal Society of Chemistry).

The electron impact reactions initiate CO_2_ dissociation which can occur through both electronic excitation and/or vibrational excitation. However, the fraction of molecules dissociating *via* each route depends on electron energy and the reduced electric field. At low values of reduced electric field (50 Td) such as the case with microwave plasma and gliding arc plasma, 90% of electron energy goes into the vibrational excitation dissociation route. At higher values of reduced electric field (300 Td) as is the case in DBD plasma, 70 to 80% of electron energy goes into electronic excitation, while only around 10% is consumed by vibrational excitation.^4^ If CO_2_ dissociates *via* vibrational excitation, only the minimum energy threshold required for the dissociation is consumed by the molecule (5.52 eV) as seen in Fig. 9 in stepwise vibrational excitation reactions.^53^ Electron excitation is the transfer of a bound electron to a more energetic, but still bound state. When the CO_2_ molecule in the reactor is excited to higher energy states *via* electron excitation, the energy provided is considerably higher than that needed for the dissociation, and therefore the excess energy is dissipated as heat.^54^ This explanation supports the observation of increased conversion and efficiency at lower temperatures, as lower temperatures could allow more energy to transfer to the vibrational levels rather than electron excitation. In conclusion, the temperature dependence of CO_2_ conversion can be explained by two competing phenomena: increasing the rate of activation *via* the vibrational ladder climbing process at lower temperatures which competes directly with a decrease of the reaction rate *via* the Arrhenius law.

**Fig. 10:**
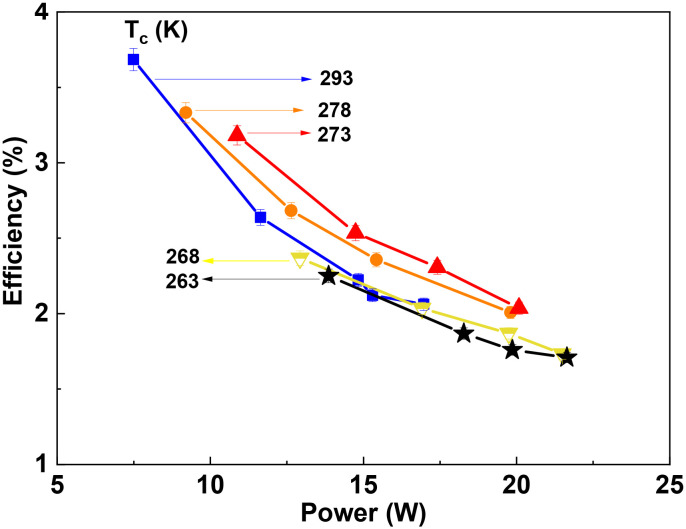
Energy efficiency as a function of input power.

Fig. 10 shows the energy efficiency as a function of power. The energy efficiency is inversely proportional to power consumption and therefore it monotonously decreases as the power increases from 10 to 20 W. When the power was kept constant, the highest energy efficiency was observed at an electrode temperature of 273 K, and either an increasing or decreasing temperature caused an obvious reduction in the energy efficiency. For example, at a power of 15 W, the highest efficiency of 2.53% was observed. However, the absolute maximum in energy efficiency of 3.8% was achieved at 293 K with the lowest power of 7.5 W. A higher power of 20 W reduced the energy efficiency towards the 1.9–2.0% range.

The energy efficiency follows the same trend as the CO_2_ conversion. Therefore, at a temperature of 273 K, the increase in conversion results in higher energy efficiency, although the absolute power consumption is higher. This suggests that an optimal temperature for energy efficiency provides a rather narrow operational window for the DBD microreactor.

A temperature controlled DBD microreactor can be a useful tool to influence the plasma discharge process and gas conversion. However, there are still challenges facing the long-term operation of DBD reactors. For example, the time required to reach the temperature setpoint and maintain the temperature at the setpoint with minimal fluctuations. This is a minor issue in microreactors where the surface area to volume ratio is large compared to larger reactors where considerable time it required to reach steady state operation. Highly effective insulation must be applied to ensure the reactor remains at the desired temperature. Advanced temperature monitoring techniques, such as TDLAS, must be used to identify the actual temperature in the reactor.

## 4. Conclusions

A plate-to-plate DBD plasma microreactor with a discharge gap of 0.50 mm has been studied in CO_2_ splitting at atmospheric pressure. The temperature of the ground electrode was maintained at a desired value in the 263–293 K temperature range during plasma operation. The effective capacitance of the dielectric layer increased by 1.5 times at a lower electrode temperature. A sharp maximum in CO_2_ conversion of 10.6% and energy efficiency was observed at an electrode temperature of 273 K corresponding to a gas temperature of 363 K. The temperature dependence of CO_2_ conversion was explained by two competing phenomena: increasing the rate of dissociation *via* the vibrational ladder climbing process at lower temperatures which competes directly with a decrease of the reaction rate *via* the Arrhenius law. The energy efficiency was inversely proportional to power consumption in the 10–20 W power range. The presence of a temperature point where both conversion and efficiency are maximized implies that reactor temperature control can be used as an effective tool to enhance the DBD reactor performance. The increase in conversion at lower temperatures is a remarkable observation. Further research is needed to explore the mechanism by which the vibrational ladder climbing dissociation promoted at lower temperatures results in higher net conversion.

## Conflicts of interest

The authors declare that they have no known competing financial interests or personal relationships that could have appeared to influence the work reported in this paper. All sources of information have been appropriately acknowledged.
